# Single-molecule chemo-mechanical unfolding reveals multiple transition state barriers in a small single-domain protein

**DOI:** 10.1038/ncomms7861

**Published:** 2015-04-17

**Authors:** Emily J. Guinn, Bharat Jagannathan, Susan Marqusee

**Affiliations:** 1 Institute for Quantitative Biosciences (QB3), University of California-Berkeley, Berkeley, California 94720-3220, USA; 2 Department of Molecular and Cell Biology, University of California-Berkeley, Berkeley, California 94720-3220, USA

## Abstract

A fundamental question in protein folding is whether proteins fold through one or multiple trajectories. While most experiments indicate a single pathway, simulations suggest proteins can fold through many parallel pathways. Here, we use a combination of chemical denaturant, mechanical force and site-directed mutations to demonstrate the presence of multiple unfolding pathways in a simple, two-state folding protein. We show that these multiple pathways have structurally different transition states, and that seemingly small changes in protein sequence and environment can strongly modulate the flux between the pathways. These results suggest that *in vivo*, the crowded cellular environment could strongly influence the mechanisms of protein folding and unfolding. Our study resolves the apparent dichotomy between experimental and theoretical studies, and highlights the advantage of using a multipronged approach to reveal the complexities of a protein's free-energy landscape.

## Abstract (web summary)

Although most protein folding experiments can be explained by a single pathway, theoretical evidence suggests the presence of multiple pathways. Here, the authors resolve this using a combination of force, chemical denaturation and mutagenesis to modulate the flux between parallel pathways.

## Graphical abstract

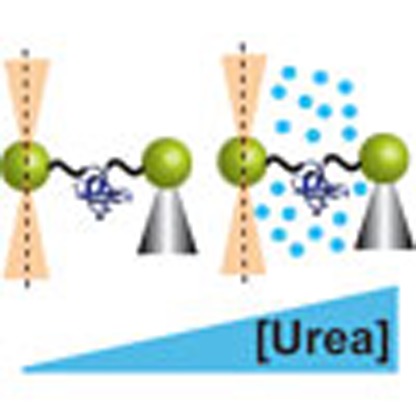

A fundamental question in protein folding is whether proteins fold through a single pathway or many parallel pathways. Theoretical studies and molecular dynamics simulations suggest that proteins access multiple folding and unfolding trajectories, describing the native state as a kinetic hub or the bottom of a funnel^1^,^2^,^3^,^4^,^5^,^6^. However, this heterogeneity of the energy landscape is rarely detected experimentally; most protein-folding experiments can be described by a simple one-dimensional reaction coordinate indicative of a single pathway^7^,^8^,^9^,^10^. It is important to distinguish between these possibilities. If parallel pathways are easily accessed, environmental changes or mutations could shift the flux between different pathways, potentially favouring a pathway prone to misfolding or aggregation. This could also mean that different regions of the protein are responsible for determining conformational lifetimes under different conditions. Moreover, parallel pathways could affect the interpretation of experiments. For instance, the effects of mutations on the kinetics of folding and unfolding are usually interpreted in terms of an effect on the height of the free-energy barrier, not a shift to a different barrier^11^.

Parallel pathways, or flux through multiple barriers, are rarely detected in experiments. The observed rate is the sum of the individual rates for all accessible pathways, so flux through multiple barriers is kinetically indistinguishable from one lower barrier. Hence, monitoring the kinetics alone cannot reveal the entire picture; experiments must also probe the structural and physicochemical characteristics of the transition state or pathway in greater detail. In some examples of experimentally observed parallel pathways, one pathway can be distinguished from the other because it populates an intermediate^12^,^13^,^14^,^15^,^16^. Parallel pathways have also been detected in repeat proteins whose symmetry allows for many energetically equivalent transition states^17^,^18^. However, for most proteins, even a detailed characterization of the folding/unfolding pathway suggests a single pathway. It is likely that in many proteins, multiple pathways are accessed, but one pathway has a much lower barrier than the others, so the dominant flux is via this pathway and the other pathways are below the experimental detection limit.

One strategy to detect parallel pathways is to perturb the energy landscape in a manner that favours one trajectory over another, potentially increasing flux through previously invisible pathways. In an elegant study, Clarke and coworkers^19^,^20^ noted a slight upward curvature in the unfolding limb of the chevron plot (natural logarithm of the unfolding rate as a function of denaturant) for a titin domain, which provided evidence for flux shifting to a parallel pathway at high denaturant concentrations. In addition to chemical denaturant^19^,^21^,^22^, other ways to perturb the energy landscape include mechanical force^23^,^24^,^25^,^26^ and point mutations^27^,^28^,^29^. Force and chemical denaturation have the benefit that they perturb the landscape in a predictable manner depending on the physicochemical features of the barriers.

Force can be applied to specific locations within a protein and the response monitored at a single-molecule level using optical tweezers^30^. We previously reported the response of the src SH3 domain to two different geometries of force application^31^. Under unzipping forces, the protein unfolds via an apparent single observable trajectory (henceforth referred to as pathway Z (for zipping)). When the protein was subjected to shearing forces, however, we obtained evidence suggesting the presence of parallel unfolding pathways (pathways S1 and S2). The relationship between these potentially different trajectories is not known, and it is also not clear how they relate to the pathway accessed in traditional, bulk experiments in the absence of force (the zero-force pathway, which we will refer to as pathway B).

To answer these questions, we now perturb the energy landscape of the src SH3 domain using a combination of force, chemical denaturant, and site-directed mutations. The effect of denaturants on mechanical unfolding has not been studied in the near-equilibrium, low-force regime of optical tweezers, although a few studies have examined co-solute effects at higher forces using atomic force microscopy^32^,^33^. Moreover, a combined analysis of force, denaturant and mutation effects is unprecedented. This experimental strategy allows us to preferentially bias different unfolding pathways and also to characterize the transition state ensemble for each pathway. We conclude that the bulk and mechanical unzipping trajectories are the same pathway. This pathway is only accessed at very low (unobservable) forces in the mechanical shearing geometry and is different than the two parallel pathways detected in the observable force range for this geometry. We also show that point mutations strongly modulate the flux between the different pathways, and we characterize the transition state ensembles using a mechanical *φ*-value analysis. Our results suggest that multiple unfolding pathways are accessed even in seemingly simple two-state proteins, but they are rarely detected because the experimental observable reports only on the pathway with dominant flux. We find that small changes in environmental conditions or point mutations can shift flux between pathways, suggesting that *in vivo*, the heterogeneous crowded cellular environment could affect protein-folding pathways. Our study helps resolve the apparent dichotomy between experimental and theoretical studies, and highlights the advantage of using a multipronged approach to reveal the complexities of a protein's free-energy landscape.

## Results

### Urea *m* ^‡^-values for different unfolding conditions

Chemical denaturant effects can be used to characterize all experimental trajectories because they can be measured in both bulk and force experiments. We denature with urea here because it is a commonly used non-electrolyte denaturant that probes burial of both amide and hydrocarbon surface in protein folding^34^. Urea unfolding *m*^‡^-values quantify the dependence of a protein's unfolding rate constant (*k*_U_) on urea via equation (1):


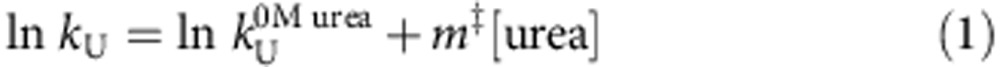


where *k*_U_^0 M urea^ is *k*_U_ in the absence of urea, and [urea] is the molar concentration of urea. Urea unfolding *m*^‡^-values are proportional to ΔASA_N->TS_, the protein surface area exposed during unfolding from the native state to the transition state; this can be used to compare the unfolding pathways of src SH3 when it is pulled across different axes and at different forces^21^,^35^.

**Figure 1:**
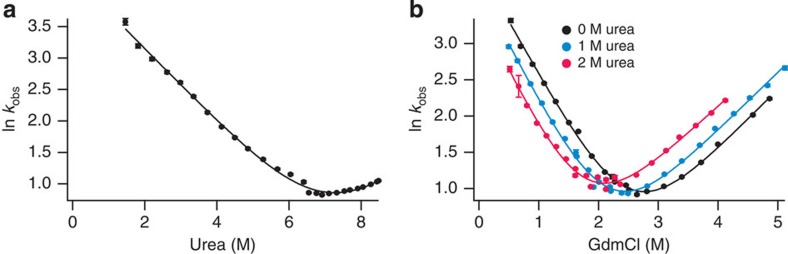
Determining bulk urea *m*^‡^-values. Bulk kinetic chevron plots for src SH3 plotting the natural logarithm of the observed relaxation rate as a function of (**a**) urea concentration or (**b**) GdmCl concentration in buffer containing 0 M (black), 1 M (blue) or 2 M urea (red). Error bars represent the s.d. of the rate measurement. These data were collected using R19C/N59C src SH3 (the unzipping variant); previous data show that *m*^‡^ for this variant is the same as *m*^‡^ for wild-type src SH3 and A7C/N59C src SH3 (the shearing variant)^31^.

First, we characterized the unfolding pathway in the absence of force. We determined the bulk urea *m*^‡^ for R19C/N59C src SH3 (the ‘unzipping' geometry variant) from kinetic chevron plots using stopped-flow fluorescence spectroscopy (Fig. 1). The concentration of urea required to completely unfold src SH3 approaches the solubility limit of urea, therefore a chevron plot of the observed rate as a function of urea (Fig. 1a) has a very short unfolding arm and the measured unfolding *m*^‡^ (0.49±0.06 M^−1^) may not be reliable. To determine *m*^‡^ under conditions where unfolding is clearly observed, we recorded three guanidinium chloride (GdmCl) dependent kinetic chevron plots in the presence of either 0, 1 or 2 M urea (Fig. 1b). For each chevron plot, we calculated the extrapolated, zero-GdmCl unfolding rate, and then fit these ln *k*_U_ values as a function of urea concentration using equation (1). The resulting *m*^‡^ (Table 1) is almost identical to the *m*^‡^ determined directly from the urea-dependent unfolding kinetics. An added benefit of this approach is that *m*^‡^ is estimated at the same low urea concentrations used in the mechanical unfolding experiments described below.

**Figure 2:**
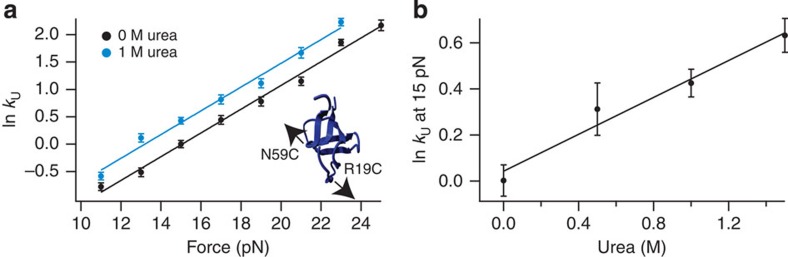
Determining urea *m*^‡^-values under unzipping forces. (**a**) The natural logarithm of the unfolding rate (s^−1^) for R19C/N59C src SH3 plotted as a function of force in buffer containing 0 M (black) and 1 M urea (blue). Inset shows the geometry along which unzipping forces were applied. (**b**) The natural logarithm of the unfolding rate as a function of urea concentration when 15 pN of force is applied in the unzipping geometry. Data were obtained using force-jump experiments in the optical tweezers and fit using the global analysis described in Methods. Each point represents the weighted average of rate measurements from at least six different protein tethers and error bars represent the s.e.

We next determined urea *m*^‡^-values for unfolding under mechanical forces. We first explored the combined effects of urea and force in the unzipping geometry (R19C/N59C src SH3) that exhibits only a single unfolding pathway (pathway Z). We measured *k*_U_ as a function of force in both 0 and 1 M urea. The resulting ln *k*_U_(*F*) versus *F* plots (Fig. 2a) were fit to the Bell model^36^:


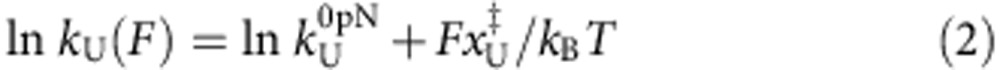


where *k*_U_^0 pN^ is the mechanical unfolding rate constant in the absence of force, *x*_U_^‡^ is the distance between the native state and the unfolding transition state along the mechanical reaction coordinate, *k*_B_ is the Boltzmann constant and *T* is the temperature. The 0 and 1 M urea plots are parallel with identical *x*_U_^‡^ values (0.89±0.03 and 0.90±0.05 nm, respectively), indicating that urea lowers the unfolding free-energy barrier without altering the unfolding pathway. In addition, because the urea *m*^‡^-value at a given force is the difference between ln *k*_U_ in 1 and 0 M urea (equation (1)), this observation also implies that the urea *m*^‡^-value is constant in the measured force range. Therefore, the data can be fit globally assuming that the urea *m*^‡^-value is force independent (see Methods for details of the global fit analysis). For the global analysis, the values of *x*_U_^‡^ and ln *k*_U_^0 pN, 0 M urea^ (the mechanical unfolding rate constant in the absence of force and urea) were fixed to the same value for the 0 and 1 M urea force-dependent data sets. The results of this analysis are summarized in Table 1.

To obtain a more detailed description of the urea *m*^‡^-value for unzipping at a single force, we also measured *k*_U_ at an applied force of 15 pN over a range of urea concentrations (Fig. 2b). The resulting plot of ln *k*_U_(*F*) versus urea concentration is linear, indicating that similar to bulk, zero-force experiments *m*^‡^ for mechanical unzipping also does not depend on the urea concentration, and hence can be determined using equation (1). This measured *m*^‡^ at 15 pN (0.40±0.05 M^−1^) is the same as the urea *m*^‡^ obtained from the global analysis of the force-dependent unfolding rates (Table 1). Therefore, the urea *m*^‡^ for a single pathway appears to be force and urea concentration independent, and can be accurately determined from data solely at 0 and 1 M urea.

**Figure 3:**
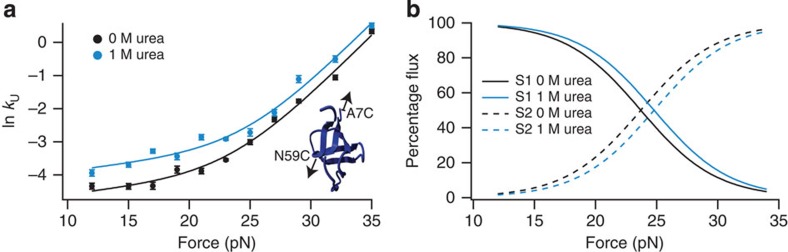
Determining urea *m*^‡^-values under shearing forces. (**a**) The natural logarithm of the unfolding rate (s^−1^) for A7C/N59C src SH3 plotted as a function of force in buffer containing 0 M (black) and 1 M urea (blue). Data were obtained using force-jump experiments in the optical tweezers and fit using the global analysis described in Methods. Each point represents the weighted average of rate measurements from at least six different protein tethers and error bars represent the s.e. Inset shows the geometry along which shearing forces were applied. (**b**) Percentage flux through pathways S1 (unbroken lines) and S2 (dotted lines) plotted as a function of force in 0 M (black) and 1 M urea (blue). Flux was calculated using equation (3) and the data from Table 1.

To determine how urea affects unfolding when parallel pathways are apparent, a similar approach was followed for the more complicated shearing geometry (A7C/N59C src SH3). We again measured *k*_U_ as a function of force in 0 and 1 M urea (Fig. 3a); in this case, however, the ln *k*_U_(*F*) versus *F* plots are not parallel to each other: urea increases the unfolding rate more significantly at lower forces than at higher forces, yielding different *m*^‡^-values in the two force regimes. This means that ΔASA_N->TS_ is different for each force regime, supporting our hypothesis of two parallel pathways (S1 and S2) that have differently structured transition states.

To calculate separate *m*^‡^-values for pathways S1 and S2, we assumed that, like unzipping, the *m*^‡^ for each shearing pathway is force and urea-concentration independent. We globally fit the 0 and 1 M urea data (see Methods for details) by fixing the ln *k*_U,1_^0 pN, 0 M urea^, ln *k*_U,2_^0 pN, 0 M urea^, *x*_U1_^‡^ and *x*_U2_^‡^ to be identical for both data sets (the subscripts 1 and 2 represent pathway S1 or S2). The data are well captured by the parallel pathways model and the resulting fit parameters are summarized in Table 1. The *x*_U_^‡^ and *m*^‡^-values for the two trajectories are significantly different from each other, again suggesting that they have structurally different transition states. Interestingly, pathway S2 has a higher *x*_U_^‡^, but lower *m*^‡^ than pathway S1. This indicates that pathway S2 has a longer distance to the transition state from the native state, but that less protein surface area is exposed in unfolding to the transition state. This result demonstrates that the end-to-end distance change and the surface area change for the unfolding of a protein are not necessarily correlated.

The parameters from the global fit can be used to calculate the flux through each pathway at any force in the range studied. The flux is simply the ratio of *k*_U_ for an individual pathway to the sum of *k*_U_ for all observed pathways:


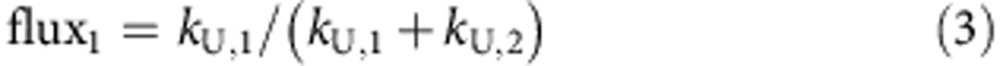


Figure 3b shows the flux through pathways S1 and S2 in 0 and 1 M urea. Under both conditions, the flux is almost entirely through pathway S1 at 12 pN; as the applied force increases, the flux contribution from trajectory S2 also increases, until it becomes completely dominant ∼35 pN. The presence of urea shifts the crossover point to a higher force. This observation suggests that for a protein unfolding via parallel pathways, small changes in solution conditions are enough to modulate the flux through the different pathways.

### Comparing all pathways

We have characterized four unfolding pathways: the bulk (zero-force) pathway (B), the unzipping pathway (Z) and the shearing pathways (S1 and S2). Are any of these the same pathway? Table 1 summarizes the *m*^‡^ and ln *k*_U_^0 pN, 0 M urea^ values measured for all trajectories observed here. Pathway B and pathway Z have very similar *m*^‡^-values, suggesting that they are unfolding through the same transition state and so are the same pathways, which we will refer to as pathway B/Z. Although it is also possible that they unfold through different transition states with similar amounts of surface area burial, the results of the mechanical *φ*-value analysis described below support the hypothesis that they unfold via the same pathway. The extrapolated ln *k*_U_^0 pN, 0 M urea^ values for pathways B and Z are not the same (pathway B extrapolates to faster unfolding in the absence of force and denaturant); however, this is likely due to the presence of the optical trap, beads and DNA handles in force spectroscopy experiments, which decrease the unfolding rate of the protein^37^.

The *m*^‡^ and ln *k*_U_^0 pN, 0 M urea^ values for pathways S1 and S2 suggest that they are different from pathway B/Z. Although the *m*^‡^ for pathway S2 is similar to that of pathway B/Z, the ln *k*_U_^0 pN, 0 M urea^ value is significantly lower, even after accounting for the effects of the optical trap and DNA handle attachment, suggesting that S2 is a distinct pathway. The *m*^‡^ for pathway S1 is significantly different from the *m*^‡^ for pathway B/Z or pathway S2, suggesting that S1 is a third unique pathway.

The presence of at least three different pathways raises the question of the potential structural differences in the corresponding transition states. While *m*^‡^-values yield ΔASA_N->TS_, they do not give any information about which regions of the protein are structured. Indeed, if different regions are structured in the transition states of the various pathways, simple mutations could modulate the flux or even alter the dominant pathway.

### Point mutations modulate the multiple unfolding pathways

We investigated the mechanical unfolding behaviour of several site-directed point variants to characterize the transition state structure of the different unfolding pathways via the *φ*-value methodology^38^. The unfolding *φ*-value indicates whether a residue is unstructured (*φ*=1) or structured (*φ*=0) in the transition state^11^,^38^. In the unzipping geometry (R19C/N59C background), most mutations cause the protein to ‘hop' between the folded and unfolded states too rapidly to reliably measure the rates on our instrument, even at very low forces (<6 pN). However, two variants exhibited slower hopping and could be reliably measured: F10I and I56A yielded unfolding *φ*-values for pathway Z (*φ*_Z_) of 0.90 and 0.55, respectively, similar to the corresponding bulk *φ*-values (*φ*_B_) of 0.88 and 0.45, respectively. The S47A variant, which does not exhibit hopping, yields *φ*_Z_=0.30 and *φ*_B_=0.20. The similarity between the mechanical and bulk *φ*-values further corroborate our hypothesis that the unzipping and zero-force, bulk-unfolding pathways are the same.

**Figure 4:**
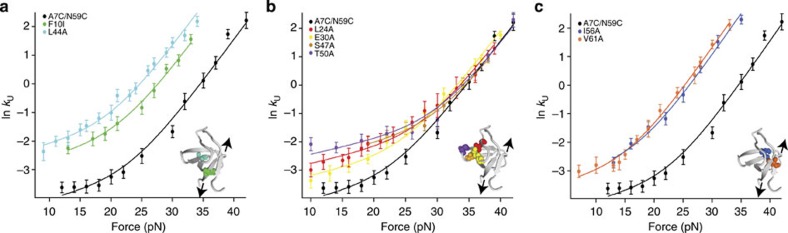
Point mutations modulate flux between pathways. Comparison of the force dependence of the unfolding rates (s^−1^) of src SH3 variants in the shearing geometry, obtained from force-jump experiments. The kinetics were recorded for at least six individual tethers, and the data shown were obtained from one such tether. Each tether was analysed separately, and there was very little variation in rates between the tethers. Some point mutations affect both pathways similarly (**a**), while others affect the two pathways differentially by lowering the barrier height of primarily either S1 (**b**) or S2 (**c**). The location of the point mutations in the protein is shown in the inset.

For the shearing geometry, all variants exhibited measurable kinetics, which could be recorded by force-jump experiments. To determine the *φ*-values in the shearing geometry (A7C/N59C background), we globally fit the data set for all the site-directed variants, using the two distances to the transition state (*x*_U1_^‡^=0.20±0.05 nm and *x*_U2_^‡^=1.20±0.04 nm) as shared variables. The point mutations can be characterized based on how they affect pathways S1 and S2. Some variants influence both pathways equally; for instance, the F10I and L44A mutations increase the unfolding rates across the entire measured force regime (Fig. 4a) by lowering the unfolding free energy barrier almost equally for both pathways S1 and S2.

Some mutations differentially affect the barrier heights of the parallel unfolding pathways, thereby changing the crossover force at which the protein switches between pathways. For instance, the T50A variant lowers the barrier for pathway S1 without affecting pathway S2; this effect manifests as an increase in the unfolding rate only at low forces, and also shifts the crossover point between pathways to higher forces (Fig. 4b). The V61A mutation has the opposite effect—it primarily lowers the barrier height of pathway S2, leading to a dramatic decrease in the crossover force (Fig. 4c). The differential effects of mutations on the parallel pathways demonstrate how seemingly small changes in a protein's sequence can favour different folding and unfolding pathways. It should be noted that the inherent complexity of the unfolding free-energy landscape for src SH3 is not evident in traditional stopped-flow experiments^39^, wherein the unfolding limbs of the GdmCl-dependent chevron plots for all variants are parallel to each other and do not show any anti-Hammond curvature (Supplementary Fig. 2, Supplementary Note 1), indicating a single dominant pathway under these conditions.

### Structure of transition state is force dependent

**Figure 5:**
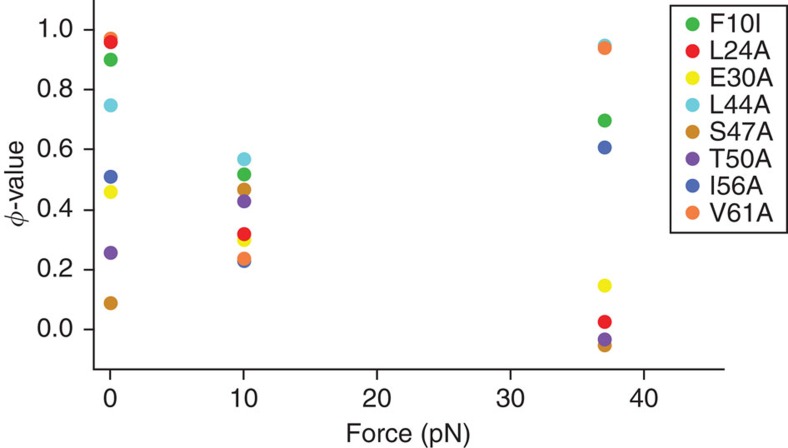
Force-dependent unfolding *φ*-values. The mechanical unfolding rates for pathways S1 and S2 were calculated using the globally fit rate at 10 pN and 37 pN, respectively. The equilibrium stability differences for the variants were estimated by bulk chevrons using stopped-flow fluorescence.

**Figure 6:**
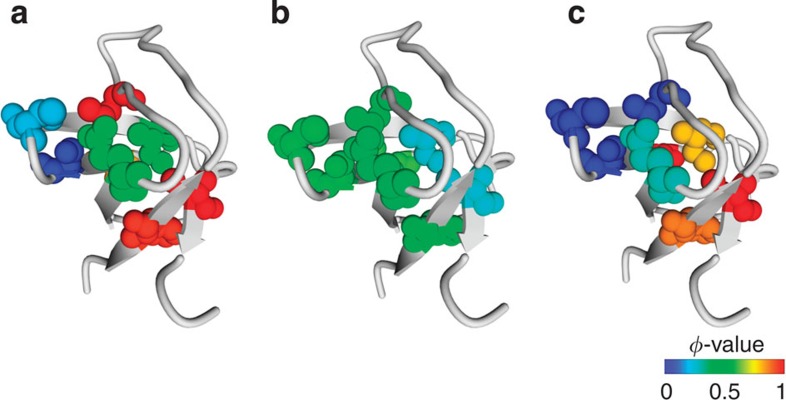
Force-dependent change in the transition state structure. The extent of structure formation in the unfolding transition states of src SH3 for (**a**) Pathway B, (**b**) Pathway S1 and (**c**) Pathway S2. The unfolding *φ*-values are represented as a heat map, ranging from *φ*=0 (completely structured, blue) to *φ*=1 (completely unstructured, red).

The calculated bulk and mechanical *φ*-values in the shearing geometry were used to map the transition state structures for Pathways S1 and S2 (Fig. 5, Table 2). Under zero-force conditions, the unfolding transition state is highly polarized, with some regions that are well structured and other regions with no structure (Fig. 6). At low forces (*F*<15 pN), all *φ*-values cluster between 0.23 and 0.57, with no extremities (Fig. 5), indicating a diffuse transition state, which can arise from partially structured residues^28^ or from the presence of multiple pathways^15^,^40^. In the latter scenario, there would be another competing pathway at low forces, and hence the *φ*-value represents an average of Pathways S1 and that pathway. Although we cannot directly observe this pathway because of the extremely slow unfolding rates at low forces, it is likely that it is the bulk-unfolding trajectory (Pathway B/Z). At zero-force, the dominant flux is through Pathway B/Z, yielding a highly polarized transition state. With increasing shearing force, the contribution from Pathway S1 increases, resulting in *φ*-values ∼0.5 due to a mixture of flux contributions from pathways with different transition state structures. This heterogeneity of the transition state at low forces cannot be detected from the ln *k*_*U*_ versus *F* plots, which yield a single distance to the transition state (*x*_U1_^‡^=0.20±0.05 nm).

At high forces (*F*>35 pN), Pathway S2 dominates, and the transition state becomes polarized again (Fig. 5). Thus, we observe a remarkable force-dependent shift in the structure of the transition state (Fig. 6). It should also be noted that although the transition state structures of the bulk pathway (Pathway B/Z) and Pathway S2 are both polarized, there are subtle differences in terms of the extent to which certain residues are structured (Table 2).

### Pulling geometry and the mechanical reaction coordinate

The ln *k*_U_^0 pN, 0 M urea^ values offer more insight into the complex energy landscape at low mechanical forces. The bulk-unfolding rates for all variants, measured in the absence of force by stopped-flow fluorescence, are significantly higher than the extrapolated, zero-force unfolding rate for pathway S1. However, it is difficult to compare the force-induced and GdmCl-induced unfolding rates directly because the latter were obtained in the absence of DNA handles, beads and the optical trap, which are known to decrease the unfolding rate^37^. With the knowledge that the mechanical ‘unzipping' and the bulk pathways are identical, we can estimate a ‘correction factor' (Δln *k*_U_^0 pN, 0 M urea^=1.48) to account for contributions of the DNA handles, beads and the optical trap.

Assuming that these instrumental factors decrease the unfolding rate similarly for all constructs, we find that in the optical trap, ln *k*_U_^0 pN, 0 M urea^ for pathway B/Z (−3.27) is higher than ln *k*_U_^0 pN, 0 M urea^ for pathway S1 or S2 (Table 1, Supplementary Fig. 3) indicating, as expected, that pathway B/Z is favoured in the absence of force and denaturant. Force along the unzipping geometry does not shift the dominant flux from pathway B/Z for any conditions studied here. Force along the shearing geometry, on the other hand, shifts the flux away from pathway B/Z so that it is not dominant at any forces studied here. At 12 pN of force in 0 M urea, the observed ln *k*_U,_ value for the shearing trajectory (−4.3) is actually lower than ln *k*_U_^0 pN, 0 M urea^ for pathway B/Z, indicating that ln *k*_U_ for pathway B/Z must decrease when shearing forces are applied, that is, a negative *x*_U_^‡^ value (Supplementary Fig. 3). Although this observation appears counterintuitive according to the Bell model, such behaviour has been previously observed in simulations on a few proteins^41^,^42^,^43^. It is also possible that the instrumental correction factor varies with pulling geometry; either way, it is apparent that ln *k*_U_ for pathway B/Z does not significantly increase with shearing forces.

Our results clearly indicate that unzipping forces favour pathway B/Z but shearing forces do not. While this may not seem intuitive, Dudko and co-workers^44^ have previously hypothesized that the reaction coordinate (the end-to-end extension in mechanical unfolding experiments) changes with the geometry of force application. Hence, the same pathway can have different *x*_U_^‡^ values depending on how it is projected on the reaction coordinate. When src SH3 unfolds to the pathway B/Z transition state, the end-to-end extension along the unzipping reaction coordinate increases more (higher *x*_U_^‡^ value) than the end-to-end extension along the shearing reaction coordinate. Our results suggest that the unfolding energy landscape is very complicated even at low perturbant concentrations, an aspect that is rarely considered in most experiments that rely on extrapolating data from high denaturant concentrations or high forces.

## Discussion

**Figure 7:**
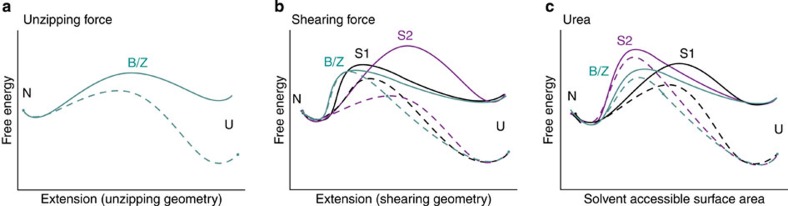
The complex energy landscape of src SH3. Schematic representation showing the features of the energy landscape of src SH3 on three different reaction coordinates. (**a**) the extension along the unzipping geometry, (**b**) the extension along the shearing geometry and (**c**) the solvent accessible surface area of src SH3. Bold lines represent the energy landscape in the absence of force and denaturant and dotted lines show the effect of force (**a**,**b**) or urea (**c**) on the energy landscape. Pathways S1 and S2 are not shown for the unzipping geometry (**a**) because we were unable to detect these pathways under unzipping forces.

By analysing combined effects of force, chemical denaturant and point mutations on unfolding of src SH3, we have identified and characterized three different pathways: pathway B/Z, pathway S1 and pathway S2 (Fig. 7). Our study resolves the apparent conflict between simulations and experiments—even though most experiments suggest a single robust pathway, we demonstrate that proteins have a choice of multiple pathways.

For most proteins under typical experimental conditions, it is likely that most of the flux is channelled through a dominant pathway that appears robust to sequence variation. We show that by perturbing with force, urea and point mutations, however, we can shift the flux between different pathways. This is relevant because in the crowded cellular environment, proteins are exposed to many perturbants that could strongly influence the choice of folding and unfolding pathways. For instance, Gruebele and co-workers^45^,^46^ find that the rate of protein folding varies with both the stage of the cell cycle and the region of the cell containing the protein. Folding in the endoplasmic reticulum even eliminates the detection of intermediates seen under other conditions. While these effects have been interpreted as a modulation of barrier heights by different cellular conditions, our results suggest that they could also be explained by a shift in flux to a different folding pathway. This is especially important because it suggests that subtle changes in the cellular environment could potentially lead to folding and unfolding along a pathogenic pathway^47^,^48^,^49^. These results also raise the possibility that proteins can evolve to fold through different pathways in different regions of the cell or stages of the cell cycle, which is supported by our observation that a single point mutation can alter the conditions where the switch between different pathways occurs. It is notable that in our experiments, the switch between parallel pathways occurs at very low, likely physiological forces, suggesting that seemingly minor mechanical perturbations inside a cell could be crucial in determining protein folding and unfolding mechanisms.

Our results also add a layer of complexity to interpretation of experimental data. Most experiments interpret the effect of a perturbant on protein-folding rates in terms of a change in the height of the rate-limiting barrier, not a shift to a parallel pathway with a different barrier. For instance, *φ*-value analysis is commonly used to characterize protein-folding transition states by assuming that point mutations only affect the height of the barrier for a single transition state^11^,^28^. If some mutations shift flux to a different pathway, the *φ*-value for that mutation is relevant to a completely different transition state.

Our multipronged approach using force, urea and point mutations has shed new light on the complexity of a protein's energy landscape. If even a simple protein like the src SH3 domain unfolds through multiple pathways, it is likely that many other proteins can also access parallel pathways even if a single dominant pathway is observed under experimental conditions. The quantitative nature of our studies, following two different reaction coordinates, provides a unique platform to test simulation methods, which should enhance the ability to connect experiments and theory.

## Methods

### Protein expression and purification

Site-directed cysteine mutations in the chicken src SH3 domain sequence were introduced using QuikChange mutagenesis. The variant proteins were expressed and purified as described previously^39^.

### Optical tweezers

DNA handles were attached to the protein as described previously^50^. The data were recorded using the optical tweezers instrument described in previous studies^24^,^51^. The optical trap is made of two coaxial, counter-propagating lasers holding a 3.2 μm, anti-digoxigenin-coated bead at the focus. This bead is tethered via the DNA–protein–DNA chimera to a 2.1 μm streptavidin-coated bead, which is held on a micropipette via suction. The micropipette is stationary, and the trapped bead is manipulated by steering the optical trap, which samples data at 1 kHz and has a spring constant of ∼0.08 pN nm^−1^.

### Bulk equilibrium and kinetic studies

Chemical denaturant melts were performed as described previously using a Horiba FloroMax-3 fluorimeter^52^. Kinetic data for the bulk chevron plots were collected on a BioLogic SFM-400/MOS 200 stopped-flow fluorescence system as described previously^52^.

### Determining bulk urea *m* ^‡^-values

The urea *m*^‡^-values were determined from a series of three GdmCl chevrons collected in buffer (100 mM Tris, 250 mM NaCl, pH 7.0) containing three different urea concentrations—0, 1 and 2 M. Each GdmCl chevron was fit to determine *k*_U_^0 M GdmCl^, the unfolding rate constant in the absence of GdmCl (but in the presence of 0, 1 or 2 M urea). The urea *m*^‡^-value was determined from the slope of a plot of these *k*_U_^0 M GdmCl^ against urea molarity.

### Determining urea *m* ^‡^-values under mechanical force

The unfolding rate of src SH3 was measured at a range of forces in both 0 and 1 M urea using force-jump experiments in the optical tweezers (typical traces shown in Supplementary Fig. 1). For each force and urea condition, at least 6 different tethers were used to collect at least 70 force-jumps; the average unfolding rate of each tether was determined, and the plotted unfolding rate was estimated as the weighted average of these rates. The data were globally analysed as described below to determine *m*^‡^-values. These experiments were performed in the same buffer (100 mM Tris, 250 mM NaCl, pH 7.0) as the bulk urea chevrons to ensure that *m*^‡^-values from bulk and single-molecule force experiments can be directly compared. A different buffer (10 mM Tris, 250 mM NaCl, pH 7.0) was used in previous experiments and in the *φ*-value analysis performed here (Supplementary Note 2). We have shown that the *φ*-values, *m*^‡^-values and *x*_U_^‡^ values are the same in both buffers (Supplementary Fig. 4; Supplementary Notes 2 and 3; Supplementary Tables 1 and 2).

### Mechanical *φ*-value analysis

The mechanical *φ*-values were calculated using previously reported methods^38^,^53^. The mechanical unfolding rates for pathways S1 and S2 were calculated using the globally fit rate at 10 pN and 37 pN, respectively. Because the attachment of the DNA handles does not affect the equilibrium stability of src SH3 (ref. 31), we estimated the stability differences for the variants by calculating the equilibrium constant using the zero-GdmCl folding and unfolding rates from bulk chevron plots (Supplementary Fig. 2, Supplementary Note 1).

### Global data analysis

In the unzipping pulling geometry, only one unfolding pathway is observed. Unfolding kinetics obtained in 0 and 1 M urea were fit using equation (4), which combines equations (1) and (2) to describe both the force and urea dependence of *k*_U_(*F*):


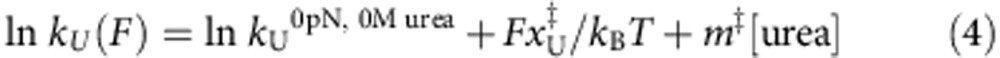


where *k*_U_^0 pN, 0 M urea^ is the mechanical unfolding rate constant in the absence of force and urea. Igor Pro v6.22a was used to globally fit both data sets to equation (4), fixing *k*_U_^0 pN, 0 M urea^, *x*_U_^‡^ and *m*^‡^ to be the same for the two data sets.

In the shearing pulling geometry, the observed *k*_*U*_ is the sum of the *k*_U_ values for pathways S1 and S2. Therefore, the data obtained at 0 and 1 M urea can be described by equation (5), which is derived from the sum of *k*_U_ values for each pathway:


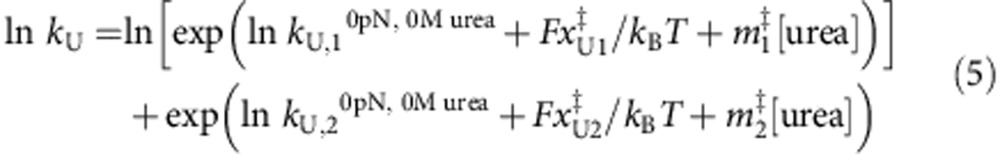


where the subscripts 1 and 2 represent pathways S1 or S2. Igor Pro v6.22a was used to globally fit the 0 and 1 M urea data to equation (5), fixing *k*_U,1_^0 pN, 0 M urea^, *k*_U,2_^0 pN, 0 M urea^, *x*_U1_^‡^, *x*_U2_^‡^, *m*^‡^_1_ and *m*^‡^_2_ to be identical for both data sets.

## Author contributions

B.J., E.J.G. and S.M. designed the research; B.J. and E.J.G. performed the research; B.J., E.J.G. and S.M. analysed the data; B.J., E.J.G. and S.M. wrote the paper.

## Additional information

**How to cite this article:** Guinn, E. J. *et al.* Single-molecule chemo-mechanical unfolding reveals multiple transition state barriers in a small single-domain protein. *Nat. Commun.* 6:6861 doi: 10.1038/ncomms7861 (2015).

## Supplementary Material

Supplementary Information Supplementary Figures 1-4, Supplementary Tables 1-2 and Supplementary Notes 1-3

## Figures and Tables

**Table 1:** Results from kinetic analysis of all unfolding pathways.

**Experiment**	**Pathway**	**Urea** ***m***^**‡**^**(M**^**−1**^)	***x***_**U**_^**‡**^ **(nm)**	**ln** ***k***_**U**_^**0 pN, 0 M urea**^
Bulk chevron	B/Z	0.46±0.04	—	−1.79±0.14
Unzipping	B/Z	0.40±0.05	0.89±0.02	−3.27±0.12
Shearing (low force)	S1	0.71±0.13	0.18±0.12	−5.04±0.42
Shearing (high force)	S2	0.36±0.15	1.52±0.12	−12.72±1.06

**Table 2:** Mechanical ***φ***-value analysis.

**Construct**	***k***_**U, S1**_ **(s**^**−1**^)	***k***_**U, S2**_ **(s**^**−1**^)	***k***_**U,B**_ **(s**^**−1**^)	**Δ*****G*** **(kcal mol**^**−1**^)	***φ***_**S1**_	***φ***_**S2**_	***φ***_**B**_
A7C/N59C	0.02	2.0	0.10	3.92	—	—	—
F10I	0.08	13.0	1.11	2.33	0.52	0.70	0.90
L24A	0.06	2.2	2.60	1.91	0.32	0.03	0.96
E30A	0.04	2.8	0.29	2.55	0.30	0.15	0.46
L44A	0.10	28.0	0.82	2.27	0.57	0.95	0.75
S47A	0.08	1.7	0.13	2.19	0.47	−0.05	0.09
T50A	0.09	1.8	0.25	1.86	0.43	−0.03	0.26
I56A	0.05	22.0	0.74	1.59	0.23	0.61	0.51
V61A	0.04	29.0	1.61	2.23	0.24	0.94	0.97

Mechanical unfolding *φ*-values measured at 10 pN (S1) and 37 pN (S2), and bulk (B) unfolding *φ*-values measured by stopped-flow fluorescence. The bulk chevron plots are shown in Supplementary Fig. 2.
