# Supplementary material for: Single-molecule chemo-mechanical unfolding reveals multiple transition state barriers in a small single-domain protein
Source: Nat Commun. 2015 Apr 17;6:6861. doi: 10.1038/ncomms7861 (PMC4410640; doi:10.1038/ncomms7861)
Supplement: Supplementary Information — Supplementary Figures 1-4, Supplementary Tables 1-2 and Supplementary Notes 1-3 [file ncomms7861-s1.pdf]

Supplementary Figure 1: Typical Mechanical Unfolding Traces. Typical unfolding traces obtained from force-jump experiments in which the optical trap is rapidly moved to a set force that is held constant until the protein unfolds from the native state (N) to the unfolded state (U). The force is then quenched to allow refolding, following which the protein is unfolded again. The sample traces shown here were obtained at 25 pN.

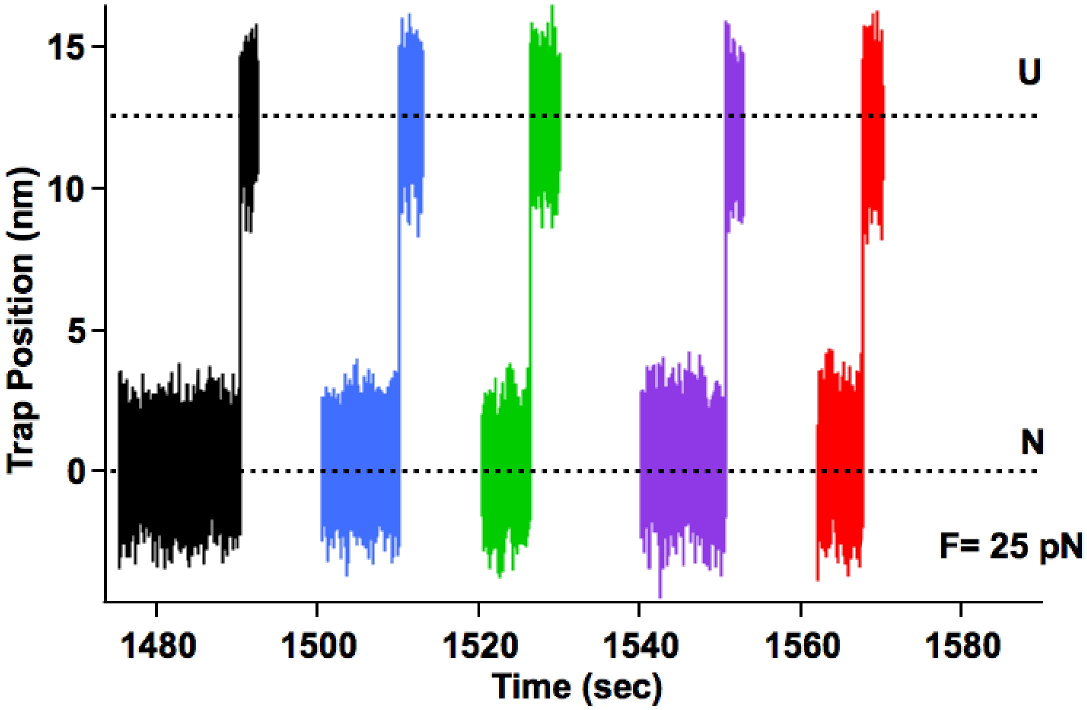

Supplementary Figure 2: Bulk Chevron Plots for Mechanical ϕ-value Analysis. Bulk kinetic chevron plots for src SH3 point variants in the shearing geometry background (A7C/N59C). The equilibrium stabilities were estimated from the zero-denaturant folding and unfolding rates.

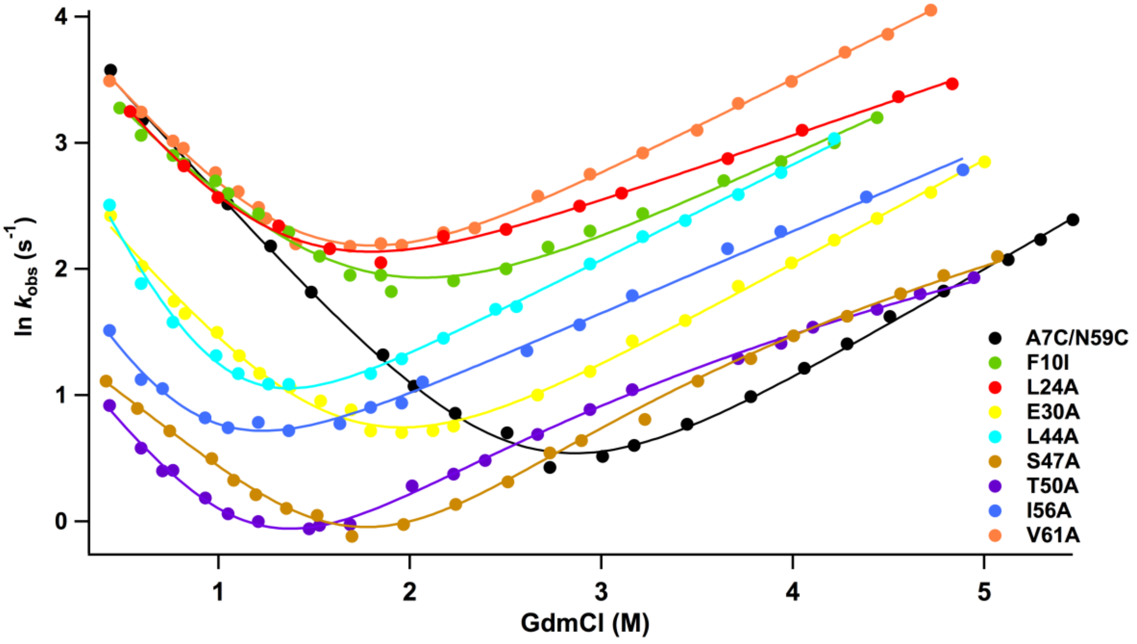

Supplementary Figure 3: Pulling Geometry and the Mechanical Reaction Coordinate. Comparison of the ln $k_U^{0pN, 0M\ urea}$ values for pathways B/Z, S1, and S2. The dotted line is intended to show that pathway B/Z likely has a negative $x_U^{\ddagger}$ trend under shearing forces but does not represent the actual slope of pathway B/Z.

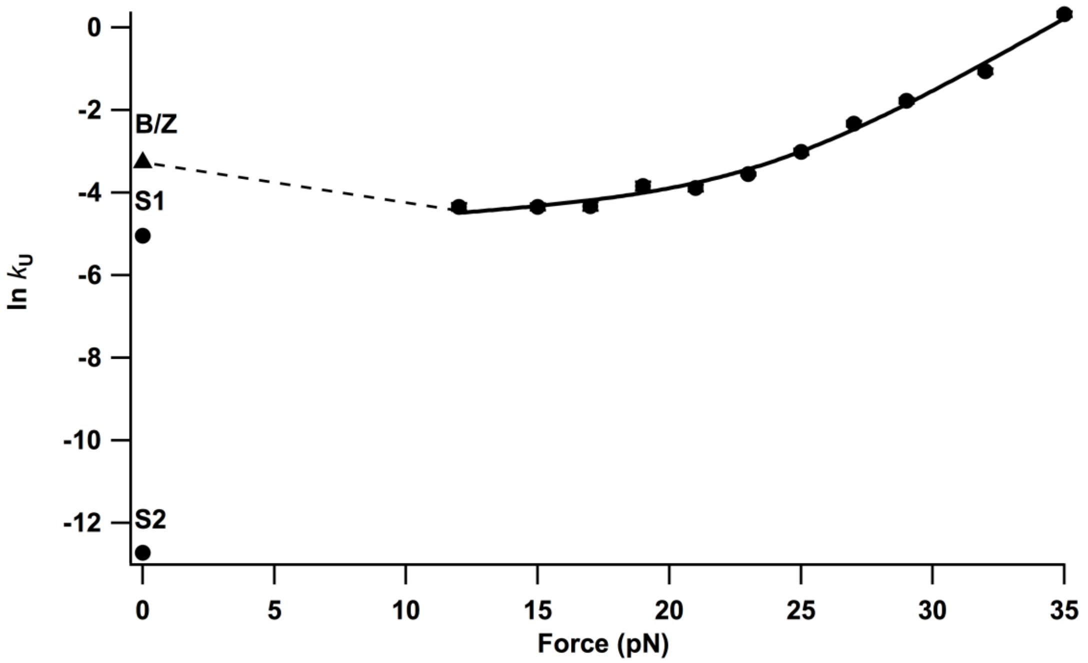

Supplementary Figure 4: Chemo-Mechanical Unfolding Analysis of the T50A Variant. The natural logarithm of the unfolding rate for the T50A variant of the shearing variant of src SH3 (A7C/N59C) when pulled in the shearing geometry as a function of force in buffer containing 0 M (black) or 1 M (blue) urea. Data were obtained using force-jump experiments in the optical tweezers and fit using the global analysis described in the supplementary information text.

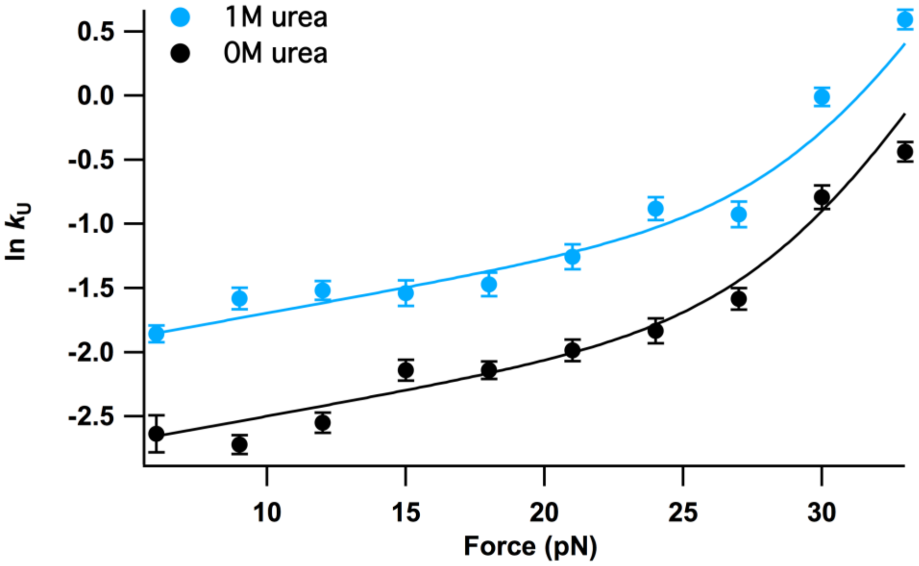

Supplementary Table 1: Comparison of different buffer conditions

	$x_U^‡$ (nm)		ln $k_U^{0pN,\ 0M\ urea}$	
Pathway	100 mM Tris	10 mM Tris	100 mM Tris	10 mM Tris
B/Z	0.89 ± 0.02	0.70 ± 0.05	-3.27 ± 0.12	-2.94 ± 0.10
S1	0.18 ± 0.12	0.20 ± 0.05	-5.04 ± 0.42	-4.55 ± 0.17
S2	1.52 ± 0.12	1.21 ± 0.14	-12.72 ± 1.06	-10.16 ± 0.38

Comparison of $x_U^‡$ and ln $k_U^{0pN,\ 0M\ urea}$ values collected in buffers containing 100 mM Tris or 10 mM Tris (both with 250 mM NaCl at pH 7.0)

Supplementary Table 2: Global data analysis

			ln $k_U^{0pN,\ 0M\ urea}$	
Pathway	Urea $m^‡$/RT (M^{-1})	$x_U^‡$ (nm)	wild-type (A7C/N59C)	T50A
S1	0.80 ± 0.07	0.16 ± 0.03	-5.03 ± 0.14	-2.90 ± 0.13
S2	0.45 ± 0.12	1.48 ± 0.08	-12.53 ± 0.64	-12.34 ± 0.71

Fitting parameters from the global analysis of kinetic data for unfolding the T50A and shearing background variants (A7C/N59C) of src SH3.

Supplementary Note 1

Chemical Unfolding Chevron Plots for ϕ-value Analysis

Supplementary Fig. 2 shows the ensemble kinetic chevron plots, obtained using stopped-flow fluorescence, that were used to estimate the bulk ϕ-values. All experiments were performed in 10 mM Tris, 250 mM NaCl, pH 7.0 buffer. It should be noted that the equilibrium stabilities (estimated from the zero-denaturant folding and unfolding rates) reported here are slightly higher than the previously published values. We have discovered that buffer exchanging src SH3 via PD-10 desalting columns (to switch from the purification buffer containing 100 mM Tris, 250 mM NaCl, 250 mM imidazole, pH 7.0 to the experimental buffer containing 10 mM Tris, 250 mM NaCl, pH 7.0) is inefficient. Instead, we have now used dialysis for performing the buffer exchange, which is more efficient and yields highly reproducible kinetic chevron plots. It should be noted that we observe conventional Hammond behavior for the S47A and T50A variants at high denaturant concentrations.

Supplementary Note 2

Comparison of Buffer Conditions

Previously published data for mechanical unfolding of src SH3, as well as the data for the mechanical ϕ-value analysis presented here were collected in buffer containing 10 mM Tris, 250 mM NaCl, pH 7.0. However, the bulk chevrons in Fig. 1, and the chemical and mechanical unfolding data in Fig. 2 and Fig. 3 were collected in 100 mM Tris, 250 mM NaCl, pH 7.0. To ensure that the different Tris concentrations used here do not affect the outcome of the experiments, we compare data for mechanical unfolding of src SH3 along shearing and unzipping trajectories in the two buffers (Supplementary Table 1). The $x_U^{\ddagger}$ values measured in the different buffers are the same within error indicating that the Tris concentration does not affect the structure of the transition state. The ln $k_U^{0pN,\ 0M\ urea}$ values, on the other hand, seem to depend on buffer

concentration indicating that Tris affects the stability of the transition state or the native state. As long as the datasets are self-consistent (all data used in the ϕ-value analysis or the chemo-mechanical analysis are collected in the same buffer), it does not matter that the ϕ-value analysis and chemo-mechanical analysis are collected in different buffers because they are still characterizing the same transition state. In fact, we show that the ϕ-values for pathways S1 and S2 in the T50A variant do not depend on which buffer is used (Supplementary Fig. 4, Supplementary Table 2).

Supplementary Note 3

Chemo-Mechanical Unfolding Analysis of the T50A Variant

As a test of the global analysis procedure used to fit chemo-mechanical shearing data, we determined $m^{‡}$ under shearing forces for the T50A variant (A7C/N59C/T50A). Assuming that the mutation does not significantly affect the structure of the transition state, the $m^{‡}$ and $x_U^{‡}$ values for T50A should be the same as those reported in Table 1. Therefore a global analysis including the T50A data should not affect these fitting parameters. Supplementary Fig. 4 shows the natural logarithm of the unfolding rate as a function of force for the T50A variant in buffer (100 mM Tris, 250 mM NaCl, pH 7.0) containing 0 M and 1 M urea. As was seen for the wild-type shearing variant (Fig. 3), the 0 M and 1 M urea trajectories are not parallel indicating that $m^{‡}$ is different at low and high forces. Because T50A increases the force at which SH3 switches between pathway S1 and pathway S2, it also increases the force at which $m^{‡}$ decreases to the lower value for pathway S2.

Assuming that T50A and the shearing background constructs of src SH3 unfold through the same pathways, both data sets can be globally fit together. We performed a global analysis where we fit all data sets (0 M and 1 M urea data for T50A and the shearing background variant) to Equation (5), fixing $x_{U1}^{‡}$, $x_{U2}^{‡}$, $m^{‡}_1$ and $m^{‡}_2$ to be identical for all data sets. Because mutation affects the stability of src SH3, we fixed $k_{U,1}^{0pN,\ 0M\ urea}$ and $k_{U,2}^{0pN,\ 0M\ urea}$ to be the same for the 0 M and 1 M urea data sets within the same mutant but not to be the same

between the different mutants. The resulting global fit parameters (Supplementary Table 2) are within error of the parameters from the original global fit to the shearing background variant (Table 1), supporting the validity of the global analysis.

These data can also be used to calculate the mechanical ϕ-value for pathways S1 and S2 for the T50A variant in buffer containing 100 mM Tris, 250 mM NaCl, pH 7.0 (unlike the ϕ-values in Table 2, which are determined in buffer containing 10 mM Tris, 250 mM NaCl, pH 7.0). The ϕ-values for A7C/N59C/T50A src SH3 determined in 100 mM Tris buffer for pathways S1 (0.59) and pathways S2 (0.05) are very similar to those reported in Table 2, which were determined in 10 mM Tris buffer. This result provides additional evidence that the Tris concentration in the buffer does not affect the structure of the transition state.
